# Retraction: Dipyrrolyl-bis-sulfonamide chromophore based probe for anion recognition

**DOI:** 10.1039/d3ra90002a

**Published:** 2023-01-13

**Authors:** Namdev V. Ghule, Sheshanath V. Bhosale, Sidhanath V. Bhosale

**Affiliations:** a Polymers and Functional Material Division, CSIR-Indian Institute of Chemical Technology Hyderabad 50060 AP India bhosale@iict.re.in; b School of Applied Sciences, RMIT University GPO Box 2476V Melbourne 3001 Victoria Australia sheshanath.bhosale@rmit.edu.au +61 399252680; c Department of Organic Chemistry, School of Chemical Sciences, North Maharashtra University Jalgaon-425001 M.S. India

## Abstract

Retraction of ‘Dipyrrolyl-bis-sulfonamide chromophore based probe for anion recognition’ by Namdev V. Ghule *et al.*, *RSC Adv.*, 2014, **4**, 27112–27115, https://doi.org/10.1039/C4RA04000G.

The Royal Society of Chemistry, with the agreement of the authors, hereby wholly retracts this *RSC Advances* article following an institutional investigation carried out by RMIT University.

Fig. 1 and S5 both contain several identical vials representing different experiments. These figures were previously corrected before the Royal Society of Chemistry was aware that this paper was the subject of an institutional investigation.

The investigation found that this paper “breached the Australian Code and RMIT Research Policy by not ensuring that conclusions are justified by the results and not responsibly disseminating research findings”. A recommendation was given that this paper is retracted.

Dr Namdev V. Ghule was fully responsible for the error that happened in the figures. All other authors, Prof. Sheshanath V. Bhosale and Dr Sidhanath V. Bhosale, are not responsible for this error.

Signed: Namdev V. Ghule, Sheshanath V. Bhosale and Sidhanath V. Bhosale

Date: 09/01/2023

## Supplementary Material

